# Supplementary figures and images for: A cercarial invadolysin interferes with the host immune response and facilitates infection establishment of Schistosoma mansoni
Source: PLoS Pathog. 2023 Feb 2;19(2):e1010884. doi: 10.1371/journal.ppat.1010884 (PMC9928134; doi:10.1371/journal.ppat.1010884)

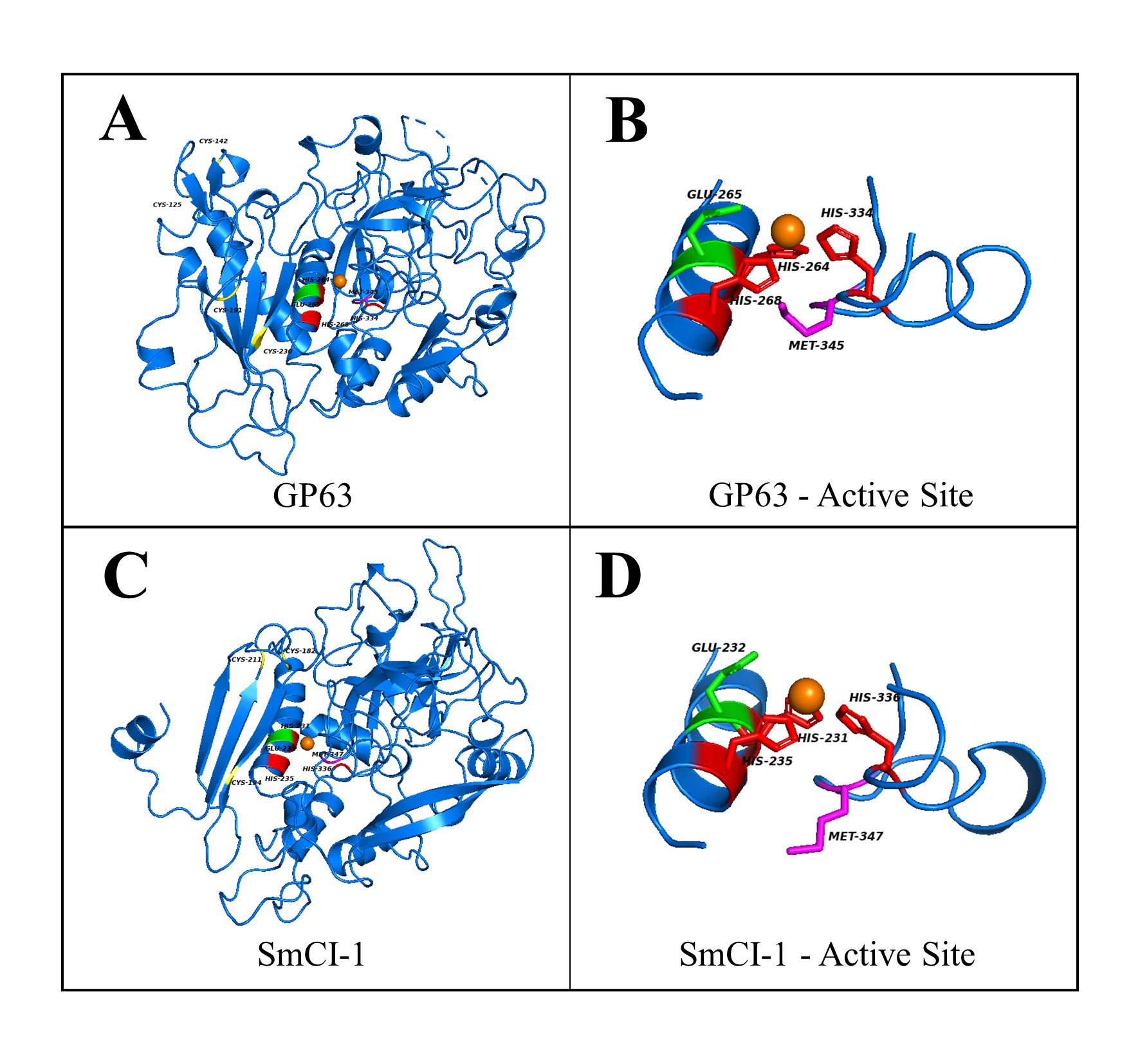

Supplement: S1 Fig — A 3D visualization of the crystal structure of GP63 (A), and a magnified view of its active site (B). The predicted structure of the full amino acid sequence of SmCI-1 is also present (C) alongside a magnified view of its active site (D). Active site histidines are presented in red, holding a zinc ion (orange ball) in position. Nucleophilic glutamic acids are marked as green, with likely met-turn methionines marked as purple. All cysteines N-terminal to the active site have been coloured yellow. (TIF) [file ppat.1010884.s001.tif]

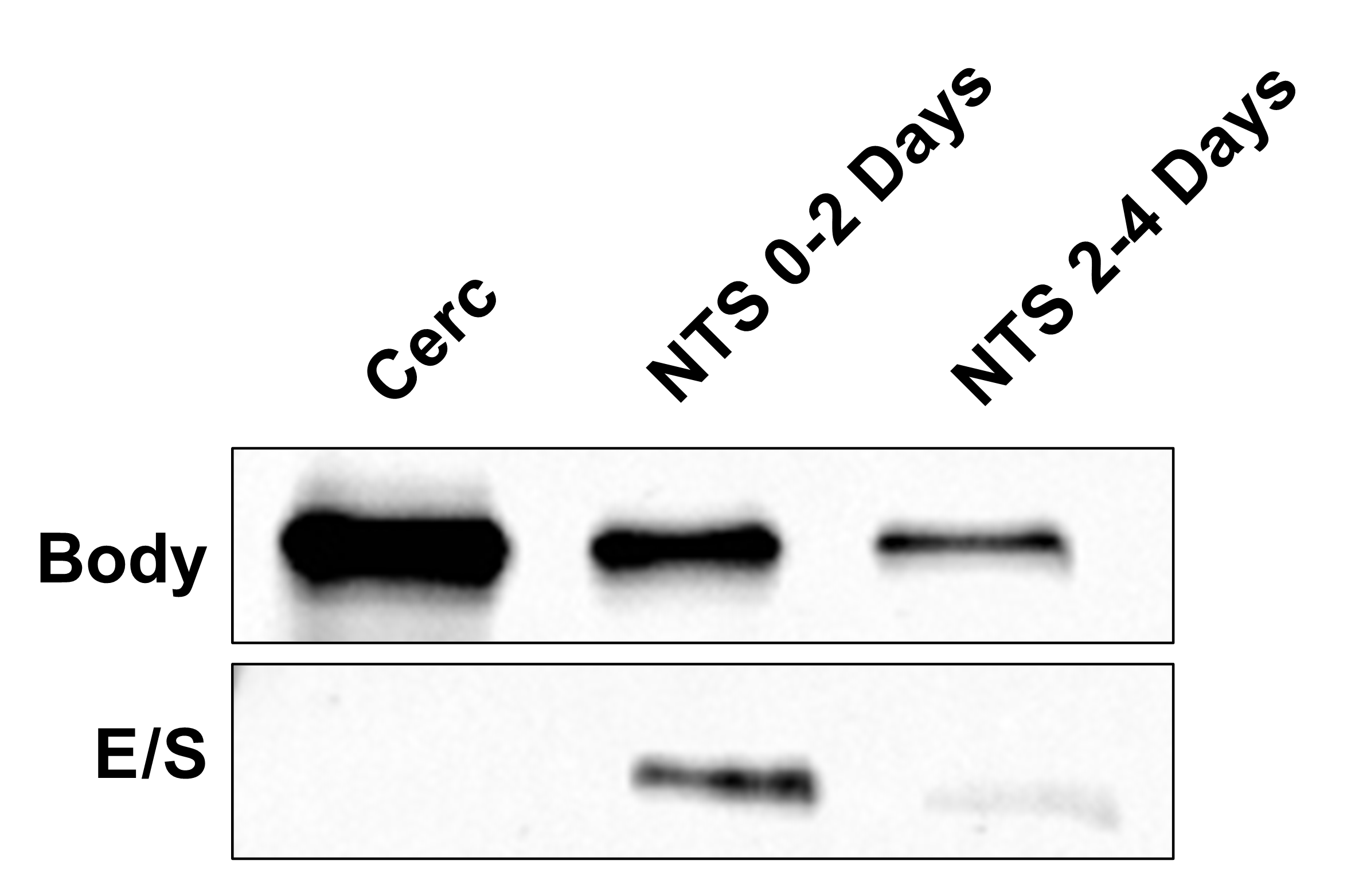

Supplement: S2 Fig — Western blot of SmCI-1 levels in untransformed cercaria, newly transformed schistosomula (NTS) cultured for two days, and NTS culture for 4 days. Accompanying E/S products obtained from the RPMI cercaria were isolated in, as well as the RPMI supernatant of 0–2 NTS and 2–4 day NTS. (TIF) [file ppat.1010884.s002.tif]

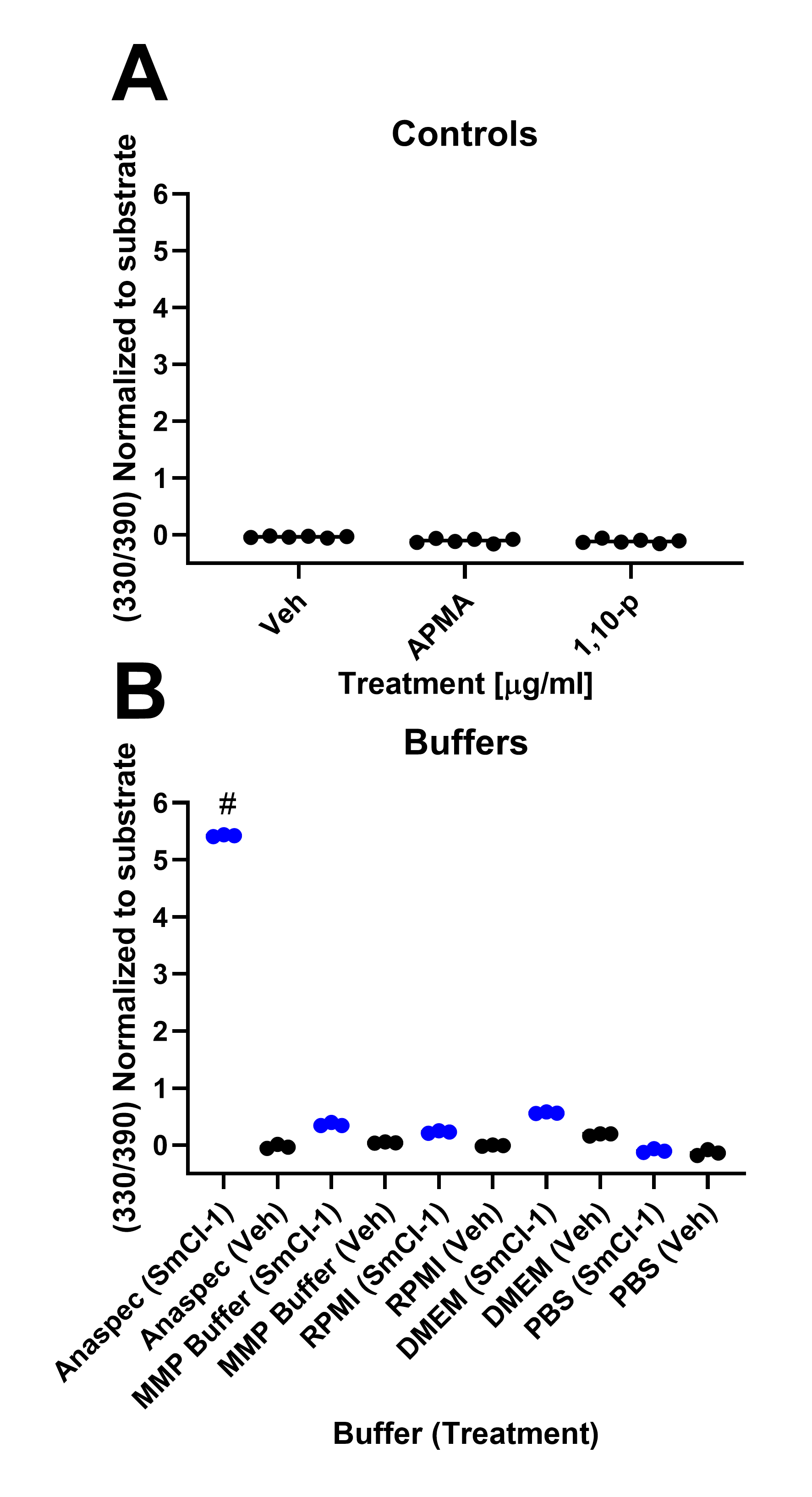

Supplement: S3 Fig — Vehicle, APMA, and 1,10-phenanthroline controls for generic MMP assay fail to vary significantly from substrate controls (A). SmCI-1 activity as measured using a generic fluorometric MMP assay in a variety of buffers (B). The highest activity is seen in the Sensolyte MMP assay provided buffer. Activity is also seen in our lab made generic MMP buffer (50mM Tris, 10mM CaCl2, 150mM NaCl, pH 7.5), as well as RPMI and DMEM. Activity is not seen in Krebs-Ringer Phosphate Buffer (KRPG) (145mM NaCl, 6mM Na3PO4, 5mM KCl, 0.5mM CaCl2, 1mM MgSO4, pH 7.4) or PBS. Statistically significant differences from vehicle only controls indicated using (*). (TIF) [file ppat.1010884.s003.tif]

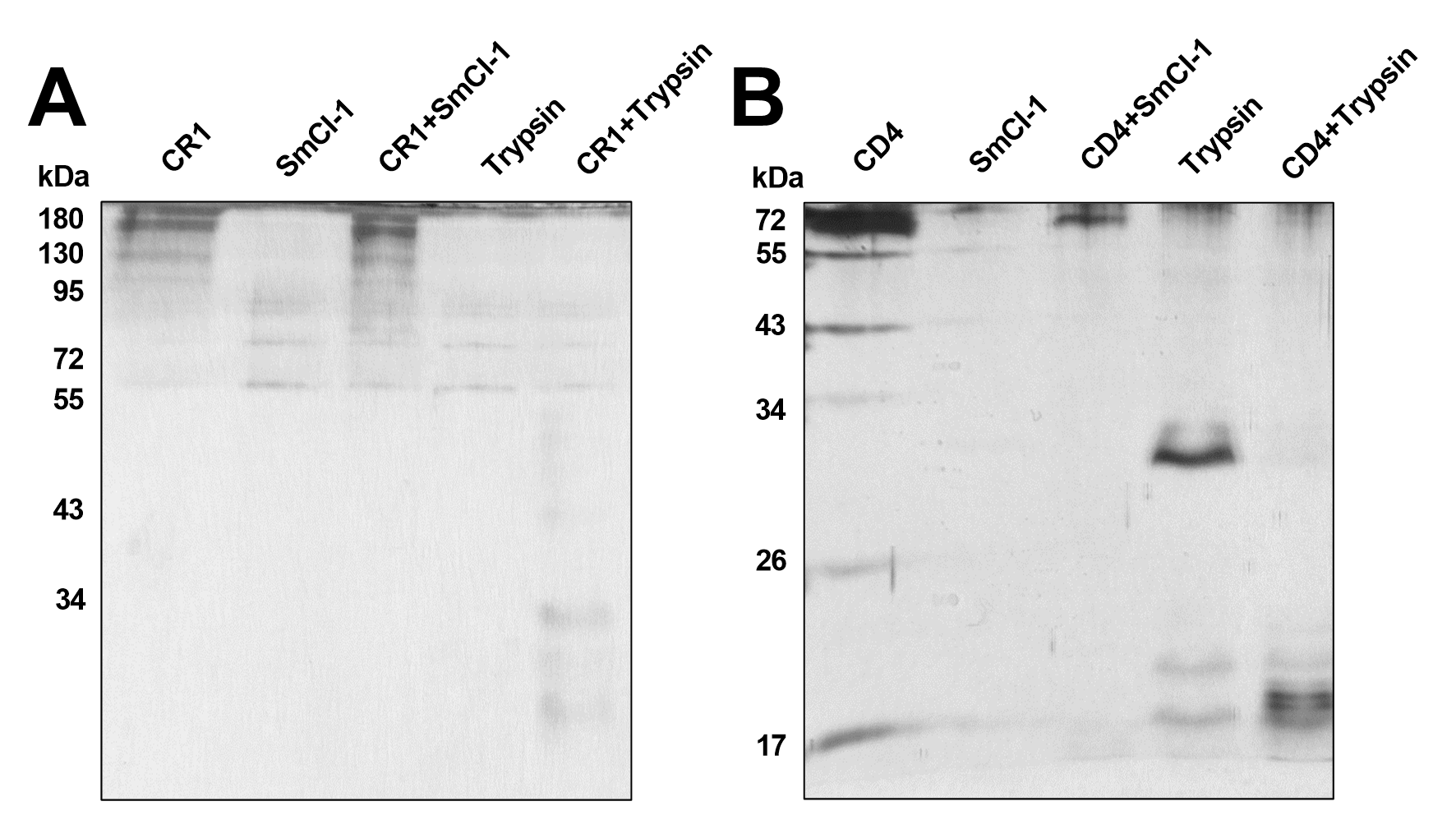

Supplement: S4 Fig — SmCI-1 fails to cleave CR1 (A), or CD4 (B). Trypsin does cleave these molecules, as evidenced by the appearance of novel bands such as the numerous bands located between 26 and 24kda when it is added to CR1, and the ~20kda bands seen when trypsin is added to CD4. (TIF) [file ppat.1010884.s004.tif]

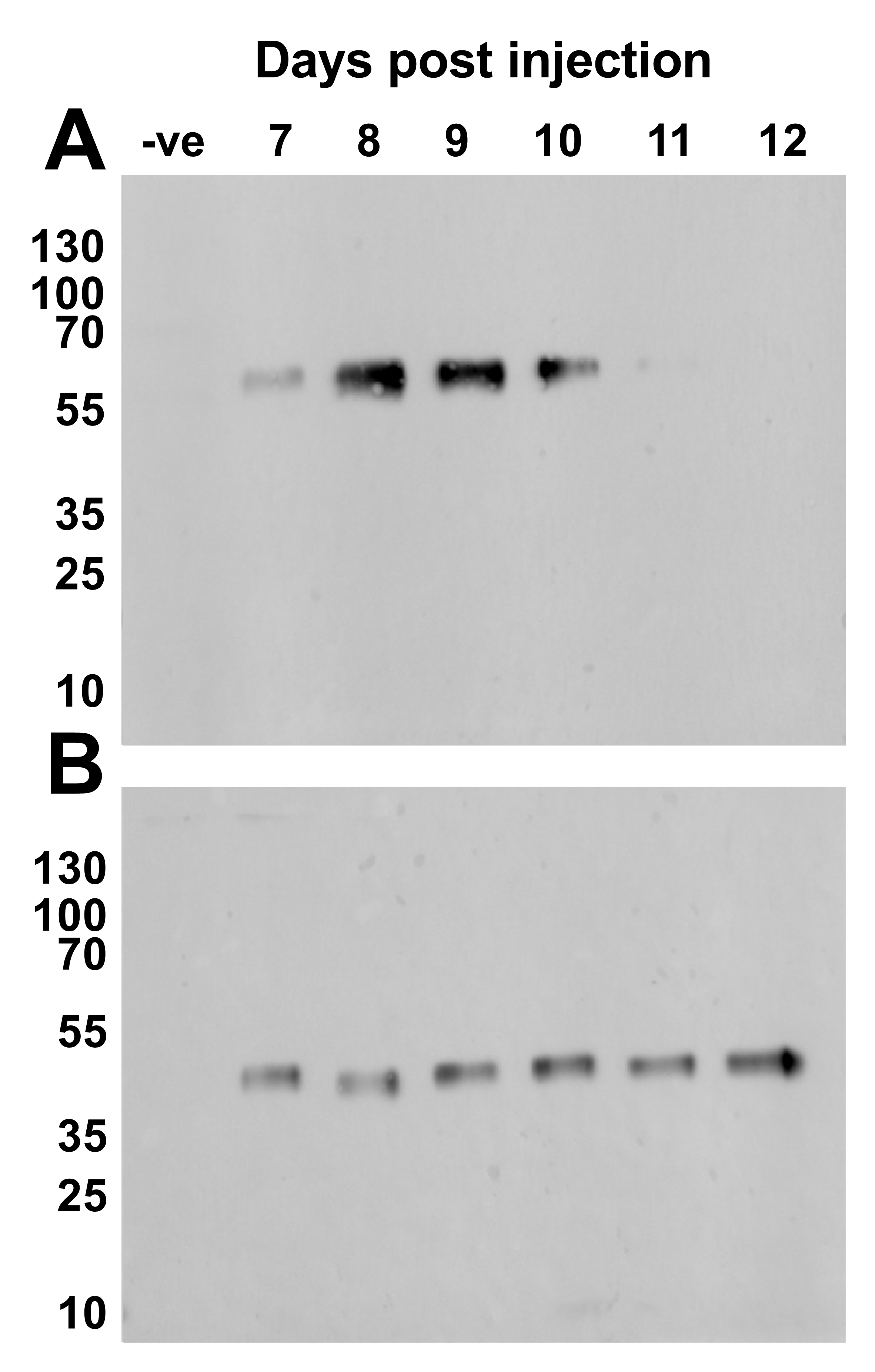

Supplement: S5 Fig — Western blot using (A) α-SmCI-1 antibody or (B) α-actin antibody on samples of 20 cercaria at varying timepoints post dsRNA construct injection. At 11 and 12 days post injection, SmCI-1 levels are significantly reduced as compared to 7–10 days post injection. (TIF) [file ppat.1010884.s005.tif]

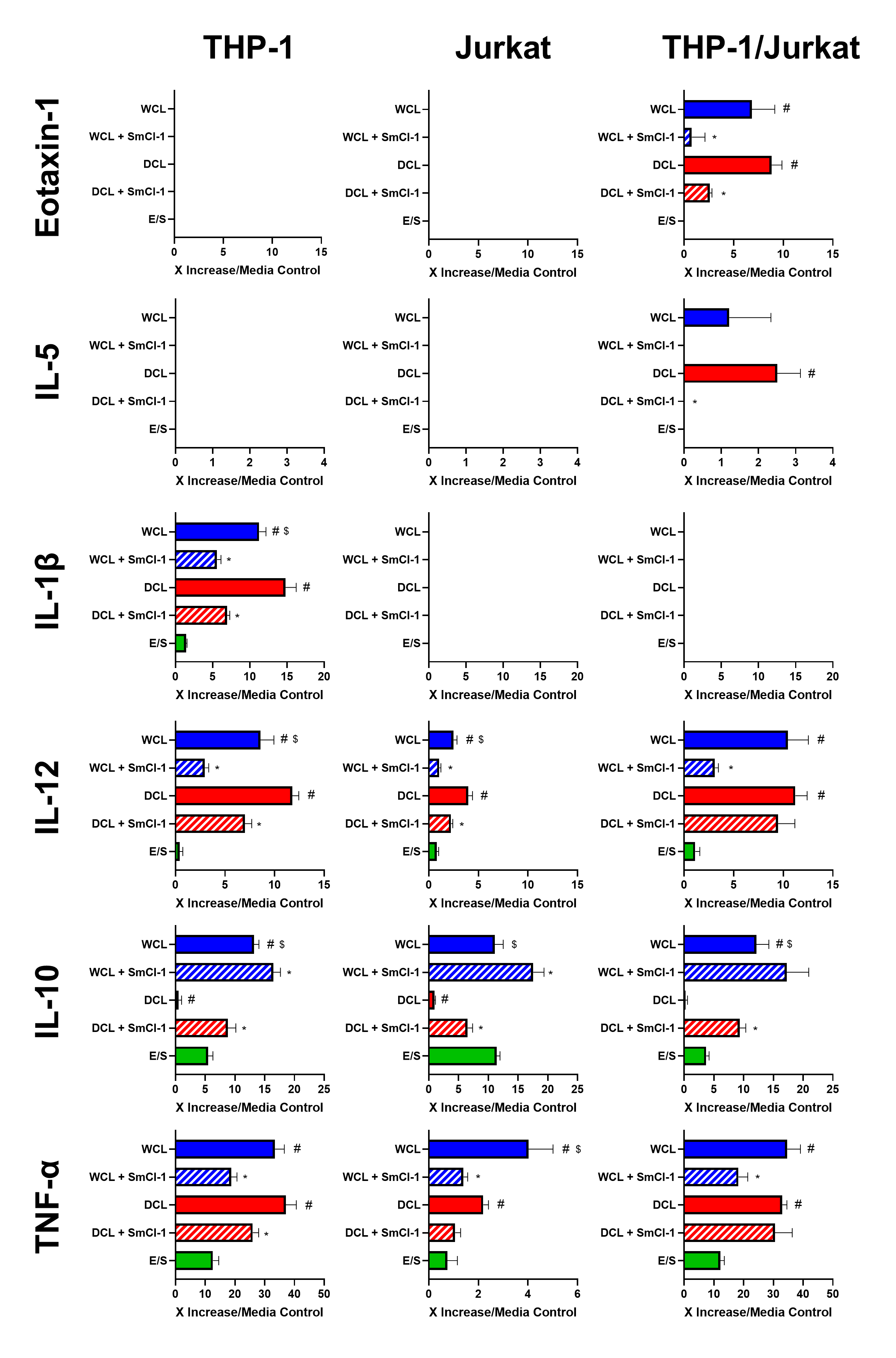

Supplement: S6 Fig — rSmCI-1 alters the production of numerous cytokines produced in THP-1 cells, Jurkat cells, and a combination of the two. Stimulation was performed using whole cercarial lysate (WCL, n = 6), drained cercarial lysate (DCL, n = 3), or cercarial excretory/secretory (E/S, n = 3) products. Those samples treated with rSmCI-1 were given 2μg/ml. (*) Indicates a significant difference for rSmCI-1 treated cells as compared to the corresponding WCL- or DCL-only treated samples. (#) Indicates WCL and DCL treated samples with significantly different levels than E/S treated samples. ($) Indicates significantly different levels of production between WCL and DCL treated samples. (TIF) [file ppat.1010884.s006.tif]
