# Supplementary material for: A cercarial invadolysin interferes with the host immune response and facilitates infection establishment of Schistosoma mansoni
Source: PLoS Pathog. 2023 Feb 2;19(2):e1010884. doi: 10.1371/journal.ppat.1010884 (PMC9928134; doi:10.1371/journal.ppat.1010884)
Supplement: S1 Table — (DOCX) [file ppat.1010884.s007.docx]

| **Individual SmCI-1 KD Oligo Sequences 5’→3’** |
| --- |
| 5’ AAGACAAUAUUCUGCAAGUGA 3’ |
| 5’ AACAUGUCAUCAGUAUUCAUG 3’ |
| 5’ AAGCUCUAUGUUAUGAUCAUC 3’ |
| 5’ AAGGAGGAACUAGCGUAUUAA 3’ |
| 5’ AAGAGAGUAGUUGGUGAUGAA 3’ |
| **Full dsRNA SmCI-1 KD sequence 5’→3’** |
| 5’AAGACAAUAUUCUGCAAGUGAAACAUGUCAUCAGUAUUCAUGAAGCUCUAUGUUAUGAUCAUCAAGGAGGAACUAGCGUAUUAAAAGAGAGUAGUUGGUGAUGAAAAGACAAUAUUCUGCAAGUGAAACAUGUCAUCAGUAUUCAUGAAGCUCUAUGUUAUGAUCAUCAAGGAGGAACUAGCGUAUUAAAAGAGAGUAGUUGGUGAUGAAAAGACAAUAUUCUGCAAGUGAAACAUGUCAUCAGUAUUCAUGAAGCUCUAUGUUAUGAUCAUCAAGGAGGAACUAGCGUAUUAAAAGAGAGUAGUUGGUGAUGAAAAGACAAUAUUCUGCAAGUGAAACAUGUCAUCAGUAUUCAUGAAGCUCUAUGUUAUGAUCAUCAAGGAGGAACUAGCGUAUUAAAAGAGAGUAGUUGGUGAUGAAAAGACAAUAUUCUGCAAGUGAAACAUGUCAUCAGUAUUCAUGAAGCUCUAUGUUAUGAUCAUCAAGGAGGAACUAGCGUAUUAAAAGAGAGUAGUUGGUGAUGAA 3’ |
| **Individual GFP KD Oligo Sequences 5’→3’** |
| 5´ CCAUCAUCUUUGAAGAAGGAACAAUCUUCUUCAAAG 3´ |
| 5´ AGGUAAUAAUACAGGACCCGGUGAUGGUCCUGUAUU 3´ |
| 5´ AUGUUGUUACUAAUGUAGCCUUGACCUACAUUAGUA 3´ |
| **Full dsRNA GFP KD sequence 5’→3’** |
| 5´CCAUCAUCUUUGAAGAAGGAACAAUCUUCUUCAAAGAGGUAAUAAUACAGGACCCGGUGAUGGUCCUGUAUUAUGUUGUUACUAAUGUAGCCUUGACCUACAUUAGUACCAUCAUCUUUGAAGAAGGAACAAUCUUCUUCAAAGAGGUAAUAAUACAGGACCCGGUGAUGGUCCUGUAUUAUGUUGUUACUAAUGUAGCCUUGACCUACAUUAGUACCAUCAUCUUUGAAGAAGGAACAAUCUUCUUCAAAGAGGUAAUAAUACAGGACCCGGUGAUGGUCCUGUAUUAUGUUGUUACUAAUGUAGCCUUGACCUACAUUAGUACCAUCAUCUUUGAAGAAGGAACAAUCUUCUUCAAAGAGGUAAUAAUACAGGACCCGGUGAUGGUCCUGUAUUAUGUUGUUACUAAUGUAGCCUUGACCUACAUUAGUA |

*Red lettering indicates nucleic acid at beginning of each oligo sequence.
