# Supplementary material for: A cercarial invadolysin interferes with the host immune response and facilitates infection establishment of Schistosoma mansoni
Source: PLoS Pathog. 2023 Feb 2;19(2):e1010884. doi: 10.1371/journal.ppat.1010884 (PMC9928134; doi:10.1371/journal.ppat.1010884)
Supplement: Attachment — Submitted filename: Review of manuscript by Hambrook and Hanington.docx [file ppat.1010884.s009.docx]

Review of manuscript by Hambrook and Hanington

This is a well written manuscript that describes a novel zinc-dependent metalloprotease that is released from the acetabular glands of larval *S. mansoni* cercariae during skin penetration to allow successful infection of the human host. Since cercariae are the first stage of the parasite to initiate the definite human host stage of the life cycle of this pathogen, it is important to discover what substances are secreted during penetration towards developing tools to block infection and, ultimately, schistosomiasis, the disease caused by this parasite.

Previous studies have shown that a variety of proteases are discharged from the acetabular glands of cercariae during skin penetration of the human host. However, this is the first study to show, comprehensively, that a zinc- dependent matrix metalloprotease, homologous to invadolysins of Leishmania makes up part of the proteolytic cocktail that is secreted from the acetabula glands. The authors also show that this novel metalloprotease, SmCI-1, plays a significant role in immunomodulation during the early infection process by dampening immune effector molecules, e.g., C3b and cytokine release in anti-inflammatory responses that favor the survival and development of the larval schistosomula parasite in the human host. As stated, though a variety of proteases have been identified in schistosomes, their functionality is less well known, making this present study timely and significant for uncovering gaps in our knowledge of what parasite proteases do in manipulating their hosts for their own survival.

Results presented in this manuscript provided clear evidence that this enzyme functions to cleave not only structural proteins (gelatin, collagen) but can upend hemolytic and cytokine cleavage activity as well.

Questions that remain for this reviewer and must be addressed in revising the manuscript are as follows:

1) Given that their data shows that this is an evolutionarily conserved protease that is homologous to an enzyme in leishmania, a protozoan, it will be interesting to know whether this metalloprotease is present in all the medically significant schistosome species.
2) With data showing the protease is related to leishmania surface protein Gp63 which is a significant lipid anchored enzyme in this parasite, would this also be the case with the schistosome homolog? It is not clear if this metalloprotease is a surface molecule in schistosomes and if it is not then how can they suggest it’s use as a vaccine candidate antigen?
3) How immunogenic is this protease? It has broad functional biological activity but is it recognized by human serum taken from patients? Antibody-dependent cell mediated immunity (ADCC) is an important mechanism in the killing of schistosomula so with results showing an effect on complement activity because of this enzyme. It will strengthen the manuscript if this long held and unequivocal mechanism of defense can be tested for antibodies raised against this enzyme.
4) The location of the metalloprotease by immunofluorescent imaging shown in Fig. 2 is not clear. In *S. mansoni* free-living cercariae, there is a post- and pre- acetabular gland, so they need to clarify which one contains this newly discovered second most abundant proteolytic enzyme in cercariae. It has previously been shown that there are 2 pairs of the pre- acetabular glands and 3 pairs of the post-acetabular glands, making 5 pairs, i.e., 10 glands in total so which of these releases SmCI-1? There is a rich literature on the structure of the cercarial acetabular glands, but none were cited in this paper.
5) The cellular organizational internal structures of this infective form of schistosomes are well documented so checking the old literature, for example, by Dorsey et al 2002 might provide a resource to address precisely where the enzyme is in cercariae.
6) The recent paper of Vondracek et al 2022 reporting the comprehensive proteomics of avian schistosome cercariae showing that relatively high levels of protein of the enzyme invadolysin exists in the absence of its corresponding transcript was also omitted in this manuscript. Several papers of shared cercarial and adult worm *S. mansoni* proteolytic surface enzymes that happen to be major antigens (diagnostic Sm31 and Sm32, Cathepsin B and hemoglobinase, respectively) were also glossed over.

Minor corrections of an editorial nature are listed below.

1) Introduction needs to be shortened. A great deal about leishmania Gp63 and SmCI-1 (lines 101-119) can be transferred to the Discussion
2) Omitted papers about the relevant publications mentioned above need to be cited.
3) Line 183 ESP were ‘collected’ for Western Blot
4) Line 187 ‘20 parasites worth’ is ambiguous. Show the concentration of protein loaded per lane.
5) Line 194 name the manufacturer of the HRP conjugate secondary antibody
6) Line 233 name the commercial source of the kit that was utilized.
7) Line 332 indicate which of the acetabular glands. There are 10 as mentioned above.
8) In Fig 2B Sm Cl-1 released in ESP appears to be smaller than 65KDa. Explain?
9) Line 514 insert ‘at the’ between immediately C-terminal
10) Line 530 change assessment to ‘results’ an insert the corresponding, Fig 2A
