# Supplementary material for: A cercarial invadolysin interferes with the host immune response and facilitates infection establishment of Schistosoma mansoni
Source: PLoS Pathog. 2023 Feb 2;19(2):e1010884. doi: 10.1371/journal.ppat.1010884 (PMC9928134; doi:10.1371/journal.ppat.1010884)
Supplement: Attachment — Submitted filename: Response to Reviewer Comments - First Review[11411].docx [file ppat.1010884.s010.docx]

**We’d like to express our thanks to the reviewers for their thoughtful comments and suggestions. We’ve included a response to each recommendation below in red.**

**Part I - Summaries**

**Reviewer #1:**

The paper describes a good characterisation of an enzyme - a metalloproteinase termed invadolysin - is stored in the acetabular gland of the infective stage of Schistosoma mansoni and based on in vitro experiments is release during the invasion of the host skin. The production of a recombinant form of the enzymes that displays proteolytic activity is well describes and it is shown to degrade various protein stubstrates. The authors also produce a mutant (needs to explain why the particular substitution was chosen) as a control. The degradation studies are performed well as is the substrate studies, although detailed kinetics are not performed. Ii general, this is a well performed characterisation of an important parasite enzyme. There is also data covering the effect of the enzyme on cytokines and on human blood mononuclear cells but these are siply observational studies without follow up functional analysis.
The manuscript needs to be reduced considerably in Introduction and Discussion, and many of the methods are standard and could be written far more succinctly. The weakness is that the paper is a basic characterisation of an enzyme and does not provide anything of particular novelty. Much of the very long Discussion repeats the results and is laboured by lots of speculation but no focused attention of nay new finding. The title gives the impression that this paper is focused on the complement system but little is done (part froma couple of non-specific assays) that would give any confidence that this protease actually plays a role in neutralising or inactivation this system. Little mention is given to this system in the Introduction, Discussion or Conclusion so why is it the major title? The effect of the protease on HBMC is interesting but not followed up in detail. Which cell population(s) is targeted.3D modelling was base on the GP63 structure because they share high homology so is it surprising that the enzyme turns out to have similarities in structure and enzyme activity to this Leishmania molecule. Many studies are performed on substrate, cytokines, cells but the data in each are preliminary and it's difficult to understand what the authors thinks of all these results and how they fit together - they Conclusion does not make anything more clear apart from stating that the enzyme has many functions displayed in a schematic that is no focus (and certainly does not indicate that complement is a major target for the enzyme).

**Reviewer #2:**

This is a well written manuscript that describes a novel zinc-dependent metalloprotease that is released from the acetabular glands of larval *S. mansoni* cercariae during skin penetration to allow successful infection of the human host. Since cercariae are the first stage of the parasite to initiate the definite human host stage of the life cycle of this pathogen, it is important to discover what substances are secreted during penetration towards developing tools to block infection and, ultimately, schistosomiasis, the disease caused by this parasite.

Previous studies have shown that a variety of proteases are discharged from the acetabular glands of cercariae during skin penetration of the human host. However, this is the first study to show, comprehensively, that a zinc- dependent matrix metalloprotease, homologous to invadolysins of Leishmania makes up part of the proteolytic cocktail that is secreted from the acetabula glands. The authors also show that this novel metalloprotease, SmCI-1, plays a significant role in immunomodulation during the early infection process by dampening immune effector molecules, e.g., C3b and cytokine release in anti-inflammatory responses that favor the survival and development of the larval schistosomula parasite in the human host. As stated, though a variety of proteases have been identified in schistosomes, their functionality is less well known, making this present study timely and significant for uncovering gaps in our knowledge of what parasite proteases do in manipulating their hosts for their own survival.

Results presented in this manuscript provided clear evidence that this enzyme functions to cleave not only structural proteins (gelatin, collagen) but can upend hemolytic and cytokine cleavage activity as well.

**Reviewer #3:**

This manuscript is original and of importance to researchers in the field of schistosome biology as well as to those interested in host-parasite interactions. As praziquantel is not effective against larval schistosome stages, an increased knowledge of cercarial biology and how they interact with the host is of particular interest in case such research leads to the identification of a cercarial molecule that could be targeted and prevent cercarial survival in the human host. This manuscript represents a comprehensive investigation of the invadolysin SmCI-1, from modelling of its structure to its effects on cytokine release; it represents a substantial amount of experimental work. The study demonstrates the presence of SmCI-1 in cercariae. SmCI-1 demonstrates weak cleavage of ECM proteins but a major finding is that it can cleave C3b suggesting an immunomodulatory role. Furthermore, it can decrease the release of select cytokines when human PBMCs were stimulated with either LPS of whole cercarial lysate.
A few weaknesses of the study are that no marker is provided in the immunocytochemistry examination of SmCI-1 for the acetabulum and/or the acetabular glands. Interestingly the acetabulum did not react with phalloidin whereas it has in a previous study. In addition, whole cercarial lysates (WCL) were used to stimulate cytokine release from human PBMCs that could be reduced by SmCI-1. However, if immunochemistry demonstrates the presence of SmCI-1 I cercariae, the WCL would also include this invadolysin. The manuscript is of a good quality though requires editing prior to publication. A few comments should also be addressed prior to publication.

**Part II – Major Issues: Key Experiments Required for Acceptance**

**Reviewer #1:**

**The paper needs to be much more focused.**

*We have taken this comment into consideration alongside other reviewer comments. While the paper still focuses on three key functions of SmCI-1 (MMP activity against structural molecules, effects on the complement system, and alteration of cytokine profiles), we have made substantial efforts to further confirm the importance of our findings in the context of parasite survival. Additionally, we have substantially reduced the amount of speculative statements made, and focused on elaborating upon the results we have obtained.*

**1. complement inactivation - discover possible targets for the invadolysin in one or all pathways. The enzyme may degrade 3C in vitro but is there a way to show this in vivo and demonstrate that this is a mean by which the parasite evades the complement system. AS mentioned in the Introduction is release of invadolysin connected with glycocalyx shedding or blocking of 3C binding the the glyoccalyx?**

*The reviewer makes an excellent point in that we had previously shown that the complement pathway was affected, but did not show that this had any tangible effect on the parasite itself. Our new data showing an ability to generate cercaria with severely depleted levels of SmCI-1 have allowed us to examine the role of SmCI-1 in surviving attack by the complement system. We demonstrate that SmCI-1 knockdown parasite viability is significantly reduced in the presence of human serum, and that parasite viability can be rescued via addition of SmCI-1, but not its catalytically inactive mutant. We also demonstrate that heat inactivation of serum results in similar rescuing of viability. This assay provides key evidence that SmCI-1 is in fact involved in protecting the developing larvae from complement mediated lysis, and that C3 cleavage is the manner by which SmCI-1 enacts this protective effect.*

**2. The protease may cleave a range of protein in vitro but are these proteins degraded in vivo - could in vivo probing with antibodies show that the parasite secrets the enzyme into the tissues. Does blocking of the enzyme prevent invasion like it is mentioned that blocking the cercarial elastase blocks skin entry.**

*Assessing the degradation of the structural and immunological proteins in vivo was predicated on numerous studies that have assessed cleavage in such a manner. See the following for examples:*

1) *Salter JP, Lim KC, Hansell E, Hsieh I, McKerrow JH. Schistosome invasion of human skin and degradation of dermal elastin are mediated by a single serine protease (2000). J Biol Chem. 275(49):38667–73.*
2) *McGwire BS, Chang KP, Engman DM. Migration through the extracellular matrix by the parasitic protozoan Leishmania is enhanced by surface metalloprotease gp63 (2003). Infect Immun. 71(2):1008–10.*

*As far as secretion into host tissues is concerned, SmCI-1 has been identified as being released from newly transformed schistosomula in our study as E/S products, a result that is consistent with the work of Curwen and associates in 2006. While other acetabular contents have been shown to be released during invasion, such as the cercarial elastase (Fishelson et al. 1992), our current animal ethics approval will not allow us to obtain skin punches in order to probe them with our polyclonal antibody. Despite this, we believe that our Western blot showing SmCI-1 in E/S products along with previously published work by other research groups is sufficient to demonstrate that the protein is released during transformation from cercaria to schistsosomula.*

*Lastly, a sincere thank you to this reviewer for suggesting that we examine whether or not SmCI-1 is important for penetration in vivo. The SmCI-1 knockdown parasites that we generated were used to determine that SmCI-1 plays a role in facilitating infection establishment in mice. While knocking down this enzyme is different from the topical inhibitor used in the cercarial elastase studies, the knockdown of SmCI-1 using dsRNA constructs was sufficient to reduce adult worm burden in infected mice.*

**3. The cellular studies need further exploration - what cells could be targeted by the enzymes and are these relevant to the in vivo situation.**

*Thank you to the reviewer for this suggestion. In order to address this comment, we have incorporated cytokine level data that we had previously obtained as exploratory results. We have included results from stimulated THP-1 cells and Jurkat cells, as well as a combination of the two. These serve as models for macrophages, T-cells, and the interaction/antigen presentation that occurs between the two. Macrophages and T-cells have been previously found at site of infection, and are involved early on during schistosome penetration of the skin (Winkle et al. 2018, Paveley et al. 2009, Kumar and Ramaswamy 1999). We elected to include these results as supplementary data for a few different reasons. The first of which is that they were obtained using a different cytokine measuring method that did not capture quantitative levels of cytokines, but rather differences in fluorescence intensity as measured via spot-blot. This meant that one to one comparison between these results and the human PBMCs was not possible. Additionally, our THP-1 + Jurkat cell samples failed to produce IL-1Beta, despite the ability of THP-1 cells to do so on their own. This occurred despite performing the assays in triplicate. Another reason we included these findings as supplementary materials is that they were performed prior to our generation of the catalytically inactive mutant form of the enzyme, further complicating comparison between this dataset and the human PBMCs. Finally, we felt that they human PBMC data would provide the best illustration of our findings, given that they do a better job of capturing the complexity of an immune response to schistosome antigens. That having been said, the addition of the human cell line data helps illustrate that the downregulation of cytokine production caused by SmCI-1 is not restricted to one cell type, but can occur in both macrophages and T cell-like cells.*

**Reviewer #2:**

Given that their data shows that this is an evolutionarily conserved protease that is homologous to an enzyme in leishmania, a protozoan, it will be interesting to know whether this metalloprotease is present in all the medically significant schistosome species.

*An excellent point. SmCI-1 has been identified proteomically in both S. mansoni and S. japonicum. Additionally, a putative ortholog been identified in the genome of S. haematobium. We have now made mention of the presence of these invadolysins in each of the three most medically important schistosome species in our introduction.*

**With data showing the protease is related to leishmania surface protein Gp63 which is a significant lipid anchored enzyme in this parasite, would this also be the case with the schistosome homolog? It is not clear if this metalloprotease is a surface molecule in schistosomes and if it is not then how can they suggest it’s use as a vaccine candidate antigen?**

*GPI prediction software such as PREDGPI does not suggest that GP63 is anchored via GPI linkage. Our immunofluorescence data does not suggest that SmCI-1 is on the surface of the parasite. We have added an explanation in our discussion as to why we believe SmCI-1 could be examined as a vaccine candidate antigen. This primarily focuses on the idea that while an immune response to SmCI-1 may not target the parasite surface, it may inhibit the immunomodulatory activity of the invadolysin, halting its protective effect against complement mediated attack as well as its anti-inflammatory properties. This would create an environment in which less parasites survive in the skin.*

**How immunogenic is this protease? It has broad functional biological activity but is it recognized by human serum taken from patients? Antibody-dependent cell mediated immunity (ADCC) is an important mechanism in the killing of schistosomula so with results showing an effect on complement activity because of this enzyme. It will strengthen the manuscript if this long held and unequivocal mechanism of defense can be tested for antibodies raised against this enzyme.**

*Regarding the immunogenicity of this protein, previous work by Crosnier and associates in 2020 revealed that antibody levels against SmCI-1 in human populations were comparatively lower than other schistosome antigens. We have added mention of this work to our discussion, and have also raised the point that the elicitation of higher antibody levels against this antigen may be beneficial in protecting against infection.*

**The location of the metalloprotease by immunofluorescent imaging shown in Fig. 2 is not clear. In *S. mansoni* free-living cercariae, there is a post- and pre- acetabular gland, so they need to clarify which one contains this newly discovered second most abundant proteolytic enzyme in cercariae. It has previously been shown that there are 2 pairs of the pre- acetabular glands and 3 pairs of the post-acetabular glands, making 5 pairs, i.e., 10 glands in total so which of these releases SmCI-1? There is a rich literature on the structure of the cercarial acetabular glands, but none were cited in this paper.The cellular organizational internal structures of this infective form of schistosomes are well documented so checking the old literature, for example, by Dorsey et al 2002 might provide a resource to address precisely where the enzyme is in cercariae.**

*Thank you to the reviewer for their recommendation in this comment. Despite performing confocal microscopy on other cercaria samples, we were unable to ascertain the precise acetabular glands from which SmCI-1 is originating. That having been said, we have incorporated additional images further confirming it’s location within the cercaria, and we feel confident that the Western blot data we provide gives us clear evidence of the release of this protein during transformation, placing it directly at the forefront of the host/parasite molecular interface. Additionally, the argument for invadolysin location within the acetabular glands is predicated upon previous work (Curwen et al, 2006, Vondracek et al, 2022). We have decided to emphasize the Vondracek paper, given it’s novel methodologies and observation that both post and pre-acetabular glands contain invadolysins.*

**The recent paper of Vondracek et al 2022 reporting the comprehensive proteomics of avian schistosome cercariae showing that relatively high levels of protein of the enzyme invadolysin exists in the absence of its corresponding transcript was also omitted in this manuscript. Several papers of shared cercarial and adult worm *S. mansoni* proteolytic surface enzymes that happen to be major antigens (diagnostic Sm31 and Sm32, Cathepsin B and hemoglobinase, respectively) were also glossed over**.

*A sincere thank you to this reviewer for reminding us of this particular finding in the Vondracek et al. 2022 paper. We have cited this paper, and mentioned in our introduction how it provides excellent evidence that such invadolysins are primarily generated during intramolluscan infection, and are not highly transcriptionally upregulated in emerging cercaria. As other reviewers indicated a need to substantially reduce the length of our manuscript, we were unable to include discussion of some of the diagnostic antigens mentioned. We did decide, however, to mention how antibody responses are higher to cathepsin B than our invadolysin in human populations.*

**Reviewer #3:**

**1) The acetabulum did not react with phalloidin in the immunochemistry (see Fig 2A) yet it did in “An Atlas for Schistosoma mansoni Organs and Life-Cycle Stages Using Cell Type-Specific Markers and Confocal Microscopy” by Collins et al., 2011. It would be useful to co-stain the cercariae in order to visualize the acetabulum as well as the acetabular glands, or co-stain the acetabular glands with PNA lectin (see Collins et al., 2011). This would support that SmCI-1 immunoreactivity is indeed in the acetabular glands. The authors could then determine whether SmCI-1 is in pre- or post-acetabular glands or both.**

*Thank you to the reviewer for their recommendation in this comment. Despite performing confocal microscopy on other cercaria samples, we were still unable to ascertain the precise acetabular glands from which SmCI-1 is originating. That having been said, we have incorporated additional images further confirming it’s presence within the cercariaand we feel confident that the western blot data we provide gives us clear evidence of the release of this protein during transformation, placing it directly at the forefront of the host/parasite molecular interface. These new images also feature phalloidin staining of the acetabulum. We were unfortunately unable to obtain PNA lectin prior to imaging.*

**Part III – Minor Issues: Editorial and Data Presentation Modifications**

**Reviewer #1:**

**1. Change title to suit data presented.**

*Edit included to put less emphasis on the role of complement degradation.*

**2. Shorten Introduction greatly**

*The introduction was cut down in size by over 25%. We have reworked some sections to retain their key points, while removing unnecessary detail.*

**3. Make Discussion more succinct.**

*Highly speculative sections of the discussion have either been eliminated or greatly cut down in size and content.*

**4. Remove modelling studies as no novel information has been gleamed from these.**

*Thank you for the recommendation. It is true that given the homology modelling technique used to generate these 3D images, we were already likely to see similarities between GP63 and SmCI-1. We have moved this image from the primary text to the supporting information, and have cut down our discussion of the structure of SmCI-1 substantially in the discussion section. We felt the figure would be appropriate for the supporting information, given that it is useful in demonstrating that SmCI-1 features the amino acids necessary for its classification as a canonical invadolysin.*

**5. Fig. 4D - please indicate a, b and Y fragments of fibrinogen.**

*Edit included in the untreated fibrinogen lane.*

**6. Fig. 5 - please add what statistical package was used.**

*Instead of indicating the statistical package used for each experiment, we have added it (Prism 9; GraphPad Software) to the statistical analysis portion of the materials and methods section.*

**Reviewer #2:**

**1. Introduction needs to be shortened. A great deal about leishmania Gp63 and SmCI-1 (lines 101-119) can be transferred to the Discussion**

*While it originally consisted of 1484 words, we have shortened the introduction to now consist of 918 words. We have reworked some sections to retain their key points, while removing unnecessary detail. For the particular lines mentioned above, we have removed mention of the individual targets, effects of invadolysins, and have instead talked about function in broad terms.*

**2. Omitted papers about the relevant publications mentioned above need to be cited**.

*We have now included mention of the 2022 Vondracek et al paper, and have also cited it.*

**3. Line 183 ESP were ‘collected’ for Western Blot**

*Edit included.*

**4. Line 187 ‘20 parasites worth’ is ambiguous. Show the concentration of protein loaded per lane.**

*While we do not have the exact protein concentration of these specific samples, we have removed the ambiguous nature of the statement. We had visually counted the number of cercaria/schistosomula under a stereomicroscope prior to the addition of reducing buffer, and therefore have elected to simply say “20 cercaria” or “20 NTS”.*

**5. Line 194 name the manufacturer of the HRP conjugate secondary antibody**

*Edit included.*

**6. Line 233 name the commercial source of the kit that was utilized.**

*Each individual kit for these assays now have the commercial source listed.*

**7. Line 332 indicate which of the acetabular glands. There are 10 as mentioned above.**

*Please see response to similar comments above. While we were unable to ascertain the exact gland in which the invadolysin was located, we have obtained more images further supporting the notion that SmCI-1 is in fact in the glands.*

**8. In Fig 2B Sm Cl-1 released in ESP appears to be smaller than 65KDa. Explain?**

*We believe that any slight discrepancies band position are solely an artifact of the Western blot transferring process. All of our Western blots recognize SmCI-1 at the same size wether it be from cercaria, NTS, or E/S products.*

**9. Line 514 insert ‘at the’ between immediately C-terminal**

*We have made changes to this sentence to make it more clear.*

**10. Line 530 change assessment to ‘results’ an insert the corresponding, Fig 2A**

*Edit included.*

**Reviewer #3**:

**1. The naming of samples reacting with the anti-SmCI-1 in western blotting (Fig. 2B) does not necessarily match terminology used lines 177-183. Fig 2B is labelled with cercariae, 24 hour cercariae, and E/S. The labelling needs to correspond more closely to the methods as it is not clear whether the E/S were gathered from transformed schistosomula that had been incubated 24 hours at 37C, or if they are from cercariae immediately post-vortexing. Also, it would help to be uniform in how to refer to transformed cercariae. It would be helpful to have a timepoint post-vortexing of cercariae that is considered full transformation of schistosomula. It would be useful to include sample of schistosomula (without supernatant) that have been cultured for 5-6 days to determine that SmCI-1 no longer present in more mature or fully transformed schistosomula.**

*In order to address this issue, we have renamed the 24 hour cercaria “newly transformed schistosomula (NTS)” and have attempted to consistently refer to schistosomula as NTS throughout the manuscript. Additionally, to address this comment’s last point, we cultured NTS and demonstrate that SmCI-1 protein levels in the parasite diminish over the span of 4 days, covering the time that the larval parasite would be vulnerable in mammalian skin.*

*We also cultured NTS for 6 days, and probed them and their E/S products for SmCI-1, revealing that the parasite does not generate new SmCI-1 protein to replace that which is released. By 4 days post transformation, little is left of the original SmCI-1. See SFigure 2. A great suggestion for an experiment.*

**2. It was very interesting to see decrease in select cytokine release when PBMCs also incubated with whole cercarial lysate (WCL). I am confused by this experimental design. I assume WCL was used to stimulate cytokine release from human PBMCs as this represents cercariae entering the human host. However, WCL was generated by sonicating cercariae 3 x 15 seconds on ice so wouldn’t the WCL (from 5 cercariae) also contain SmCI-1? If so, then the reduction of cytokine release when also incubated with SmCI-1 is due to a high concentration of SmCI-1 that is not physiologically relevant. If WCL does not contain SmCI-1 the authors should clarify that and explain why. Furthermore, the total protein content of WCL should be included.**

*A great point regarding the stimulant we used in this system. You are correct in assuming that WCL would have endogenous SmCI-1 present. We merely wanted to observe any additive effects that the addition of the recombinant would elicit. That having been said, in order to address these concerns, we have added data from drained cercaria lysate (DCL) stimulated cells. These parasites had been transformed into NTS, and had expulsed E/S products. This theoretically removes the anti-inflammatory components of the acetabular glands, while retaining the immunostimulatory components of the parasite’s body. We observed DCL being more stimulatory for several cytokines than WCL, which is consistent with WCL possessing anti-inflammatory factors that DCL does not. Additionally, rSmCI-1 addition to DCL stimulated cells resulted in key cytokines being produced at levels similar to WCL-only stimulated cells, consistent with rSmCI-1 being capable of reducing inflammation. Regarding the physiological relevant of our rSmCI-1 concentrations, we elected to use 2ug/ml given that it is similar to some concentrations used when functionally characterizing other S. mansoni cercarial factors such as SmCE and Sm16.*

**3. Ref 32 shows that Smp_090100.1 is more abundant in germ balls than cercariae as the authors refer to line 550-2. Might this suggest that SmCI-1 plays a role in emergence from snail? I think the authors should discuss the possible role(s) of SmCI-1 in the snail.**

*Thank you to the reviewer for this suggestion. Several factors render it unlikely for SmCI-1 to be highly involved in emergence from the snail. The first is that snail immune mediated destruction primarily occurs during the first 48 hours post infection with a miracidium. By the time cercaria are being shed, sporocysts can occupy up to 40% of the snail’s biomass, and immune mediated destruction is highly unlikely. Specifically regarding emergence, given the localization of SmCI-1 in the acetabular glands, we are unaware of any literature suggesting that acetabular gland contents are released in the intermediate host. In fact, the majority of authors argue that they are only released during penetration into mammalian skin (example - Knudsen et al. 2005). While a role for SmCI-1 in emergence from the snail could theoretically exist, the aforementioned facts, accompanied by the reality that cercaria emerge from daughter sporocysts and are shed immediately after doing so, suggests that the predominant role of the invadolysin lies in facilitating infection of the mammalian host.*

Editing notes
**Line 4 if both authors from same institution * not necessary**

*Edit included.*

**Line 98 should also cite paper where referred to as Smp_090100.1 (ref 32).**

*Edit included.*

**If in ref 26 SmCI-1 = SmPepM8 please include this nomenclature**

*Edit included.*

**Lines 106-7 should leishmania be Leishmania ?**

*Edit included.*

**Line 118 period missing after (54)**

*Edit included.*

**Line 148 clarify whether genetic sequence synthesized commercially or via PCR in house. Please include details of company for expression vector**

*Edit included.*

**Line 149 Please include details of how transfection performed**

*We have now included both the transfection reagent and protocol.*

**Line 153 It would be helpful to state the importance of this particular mutation to function. This is addressed in the conclusions, but it would be helpful to include briefly in methods. Please clarify if mutant protein purified the same way as the WT was.**

*Edit included.*

**Line 155 Please replace “Acetabular genetic sequence” with SmCI-1 for the purposes of uniformity and clarity**

*Edit included.*

**Line 158 Kanamycin does not need “K” if not first word of sentence**

*Edit included.*

**Please be uniform in use of Thermo Fisher Scientific, varies throughout methods**

*Edit included.*

**Line 169 replace “anti-Sm-CI-1” with anti-SmCI-1 for uniformity
Please clarify if antibody was affinity purified using recombinant protein**

*Edit included.*

**Line 171 Clarify how many snails were exposed to miracidia**

*Edit included.*

**Line 174 Clarify whether snails placed in artificial pond water for cercarial emergence**

*Edit included.*

**Line 175 Please include length of vortex step**

*Edit included.*

**Line 176 Please include temperature for centrifugation step**

*Edit included.*

**Line 179 What will these supernatants be used for?**

*Edit included.*

**Line 182 Please clarify whether E/S products were filtered and/or concentrated before use**

*Edit included.*

**Line 187 Please clarify quantification of ~ 20 parasites per lane, i.e., does it mean ~ 20 untransformed cercariae, ~ 20 schistosomula, and E/S from ~ 20 schistosomula?**

*Edit included.*

**Line 188 Please replace “blotted” with “transferred” and include details of transfer**

*Edit included.*

**Line 190 Bovine Serum Albumin does not require uppercase letters**

*Edit included.*

**Line 191 Please include the final concentration of the primary antibody in ug/ml**

*Edit included.*

**Line 194 Please include company secondary antibody sourced from**

*Edit included.*

**Line 201 Please be uniform in use of “cercariae” for plural of cercaria, most other times in manuscript given as cercaria**
*Edit included.*

**Line 201 phosphate does not require uppercase p**
*Edit included.*

**Line 205 Please include final concentration of antibody in μg/ml**

*Edit included.*

**Line 224 Please change “difference” to differences (lines 241 and 247 also)**

*Edit included.*

**Line 255 Please include software used for statistical analyses**

*Edit included.*

**Lines 228-9 PH should be pH**

*Edit included.*

**Line 249 Please change “whom” to which**

*Edit included.*

**Line 251 Can abbreviate “micrograms” to μg**

*Edit included.*

**Line 254 Why is mutant Sm-CI1 not included? Please be consistent in wording of Sm-CI1 as sometimes comes with “r” in front and other times does not**

*Edit included. rSmCI-1 is used in methods and results, while general discussion of the molecule is now simply done using SmCI-1. Regarding why the mutant form of SmCI-1 was not included in substrate degradation assays, we elected not to include it given that our generic MMP assay revealed that it had no proteolytic activity, and was therefore not going to cleave any substrates.*

**Line 265 Need punctuation between ingredients of buffer**

*Edit included.*

**Line 269-70 please remove “in”**

*Edit included.*

**Line 285 M of mM typically not italicized**

*Edit included.*

**Line 289 can replace 9g NaCl in 1L with 0.9% NaCl**

*Edit included.*

**Line 296 Should bio-techne be Bio-Techne?**

*Edit included.*

**Line 307 please insert comma after rMutSm-CI1**

*Edit included.*

**Line 308 please include final concentration of antibody used**

*Edit included.*

**In controls shown in Fig 2A in methods section
The figure legend for 2B figure states “SmCI-1 is expelled from the cercaria during the initial stages of maturation into schistosomula that occur in human skin”. I would consider this statement a conclusion that should be given in said section rather than in the results. If kept in results a description of the WB should be given first i.e., western blot showing Sm-CI-1 immunoreactivity in…..**

*Edit included.*

**Lines 436-8 state “ Eotaxin-1 (1138±301 pg/ml), IL-5 (332±42 437 pg/ml), IL-1β (394±102 pg/ml), and IL-12 (189±21pg/ml) levels all failed to differ from the WCL only treated cells in a significant manner”. Is this line supposed to state that the mutant SmCI-1 does not cause a significant reduction vs WCL stimulation?**

*Edit included. This sentence was meant to convey that no significant differences from WCL treated samples were seen when cells were treated with the mutant. We have added this to the text.*

**Line 442 does “stimmed” mean stimulated? If so please change to latter**

*Edit included.*

**Lines 458-9 “The effect of rSmCI-1 on IL-10 levels features an increase is shown for both cells exposed to WCL”. Would change wording as does not flow well, perhaps “is shown” should be omitted.**

*Edit included.*

**Line 607 “which have display” should be should have displayed**

*Edit included.*

**Line 615 add “was” after “CD4”**

*Edit included.*

**Line 623 “factor” should be plural**

*Edit included.*

**Line 641 eotaxin starts with lower case e but it is uppercase throughout**
*Edit included.*

**Line 646 please give dendritic cells in full**

*Edit included.*

**Line 650 “suggest” should be suggests**

*Edit included.*

**Line 661 “mediate” should be mediated**

*Edit included.*

**Several references contained various formatting issues or lacked italicization of scientific names: 14, 15, 43, 44, 48, 52, 55, 64, 67, 68, 70, 76, 77**
*Edit included.*

**For supplementary table 1 please clarify if acetabular gland contents are the E/S from transforming cercariae.**

*Edit included. Now just says E/S products.*
